# Case report: Two sisters with a germline *CHEK2* variant and distinct endocrine neoplasias

**DOI:** 10.3389/fendo.2022.1024108

**Published:** 2022-11-07

**Authors:** Raphaelle D. Vallera, Yanli Ding, Kimmo J. Hatanpaa, Justin A. Bishop, Sasan Mirfakhraee, Abdel A. Alli, Sergei G. Tevosian, Mouna Tabebi, Oliver Gimm, Peter Söderkvist, Cynthia Estrada-Zuniga, Patricia L. M. Dahia, Hans K. Ghayee

**Affiliations:** ^1^ Department of Medicine, Division of Endocrinology, Baylor Scott & White Health, Dallas, TX, United States; ^2^ Department of Pathology, University of Texas Health Science Center at San Antonio, San Antonio, TX, United States; ^3^ Department of Pathology, University of Texas Southwestern Medical Center, Dallas, TX, United States; ^4^ Department of Medicine, Division of Endocrinology, University of Texas Southwestern Medical Center, Dallas, TX, United States; ^5^ Department of Physiology and Functional Genomics, University of Florida, Gainesville, FL, United States; ^6^ Department of Physiological Sciences, University of Florida, Gainesville, FL, United States; ^7^ Department of Surgery and Department of Biomedical and Clinical Sciences (BKV), Linköping University, Linköping, Sweden; ^8^ Division of Cell Biology, Department of Biomedical and Clinical Sciences (BKV), Linköping University, Linköping, Sweden; ^9^ Clinical Genomics Linköping, Science for Life Laboratory, Linköping University, Linköping, Sweden; ^10^ Department of Medicine, Division of Hematology and Medical Oncology, Mays Cancer Center, University of Texas Health Science Center at San Antonio, San Antonio, TX, United States; ^11^ Department of Medicine, Division of Endocrinology & Metabolism, University of Florida, Malcom Randall VA Medical Center, Gainesville, FL, United States

**Keywords:** CHEK2, PNET, PCC, NF1, germline, somatic, tumor

## Abstract

Genetic testing has become the standard of care for many disease states. As a result, physicians treating patients who have tumors often rely on germline genetic testing results for making clinical decisions. Cases of two sisters carrying a germline *CHEK2* variant are highlighted whereby possible other genetic drivers were discovered on tumor analysis*. CHEK2* (also referred to as CHK2) loss of function has been firmly associated with breast cancer development. In this case report, two siblings with a germline *CHEK2* mutation also had distinct endocrine tumors. Pituitary adenoma and pancreatic neuroendocrine tumor (PNET) was found in the first sibling and pheochromocytoma (PCC) discovered in the second sibling. Although pituitary adenomas, PNETs, and PCC have been associated with *NF1* gene mutations, the second sister with a PCC did have proven germline *CHEK2* with a pathogenic somatic *NF1* mutation. We highlight the clinical point that unless the tumor is sequenced, the real driver mutation that is causing the patient’s tumor may remain unknown.

## Introduction

With advances in genetics, more patients are being referred to genetic counseling to help identify pathogenic mutations they may be harboring that could affect their own management and identify at-risk family members. However, even when a germline mutation is discovered, it may not always be the driver mutation causing the patient’s tumors. In this case series, we describe two sisters with a germline *CHEK2* mutation in addition to endocrine tumors that may have driver somatic mutations that could give rise to endocrine neoplasms. *CHEK*2 encodes for a serine threonine kinase involved in the response to DNA damage. Loss of CHEK2 function has been documented in breast cancer (1, 2) colon cancer, renal cell carcinoma (RCC), prostate cancer, ovarian cancer, and some cases of Li-Fraumeni syndrome (3, 4), a familial syndrome more commonly associated with the tumor suppressor gene *p53.* Patients with Li-Fraumeni syndrome are at risk for breast cancer, soft tissue sarcomas, and adrenal cortical carcinoma (ACC). When CHEK2 is activated, it inhibits CDC25C phosphatase, thereby stabilizing the p53 tumor suppressor protein and causing cell cycle arrest in G1 (5). Recently, *CHEK2* mutations were described in the context of endocrine cancer: one patient with a germline *CHEK2* mutation with ACC was reported (6), and a pathogenic variant of the gene was documented in a patient with multiple endocrine gland tumors (7). Moreover, associations between *CHEK2* and pancreatic neuroendocrine tumors (PNETs) have been detected from whole genome sequencing (8). Here we describe a patient with a history of colon polyps, pituitary adenoma, PNET, and a *CHEK2* c.1100delC germline mutation (9, 10). The index patient’s sister, who had a history of pheochromocytoma (PCC) was also found to carry the same germline mutation in *CHEK2* and a known pathogenic *NF1* somatic mutation (p.Lys1444Glu) (11–13).

PCCs arise from the chromaffin cells of the adrenal medulla and have high heritability with a large roster of germline or somatic mutations. PCCs associated with germline gene mutations are currently categorized into three main clusters: pseudohypoxia (Cluster I), kinase signaling (Cluster II), and the third cluster characterized by expression of the *CSDE1* and *UBTF-MAML3* genes associated with an active Wnt signaling pathway (Cluster III) (14). Germline mutations in the neurofibromatosis gene (*NF1*) belong to Cluster II and account for approximately 3% of all PCC cases (15, 16), while the frequency of somatic mutations in PCC is estimated to be 20-30%; both germline and somatic *NF1* mutations have been amply described in PCC (17–19).

## Objective

Here we report that both sisters have a germline *CHEK2* mutation, and in at least one of the tumors, a somatic mutation that may be the driver for their endocrine neoplasias was identified in the *NF1* gene. The connection between *NF1* and *CHEK2* mutations in PCC remains to be explored. Although clinical relevance of *CHEK2* mutations in these cases cannot be confirmed, we hypothesize that it may play a role in both cases. This report illustrates how sequencing the actual tumor can elicit the driver mutation that germline testing alone would have been unable to identify.

## Description of cases and diagnostic assessment

### Case 1

**Figure 1:**
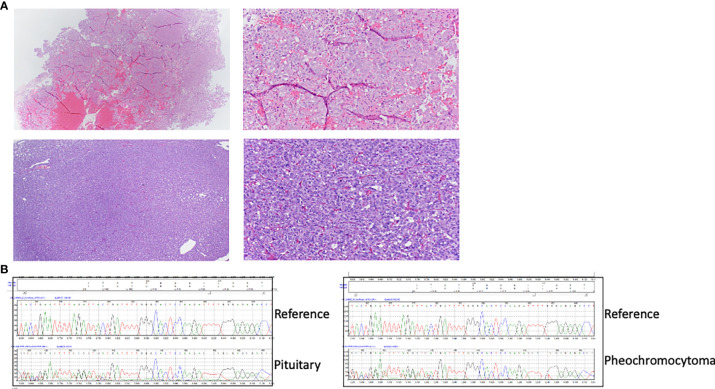
**(A)** HE images of the proband’s pituitary adenoma (top) and her sister’s pheochromocytoma (bottom). Images on the left were taken at lower (40X) magnification and those on the right at higher (100X) magnification. **(B)** *CHEK2* sequence traces from DNA obtained from the proband’s pituitary adenoma (top) and the sister’s pheochromocytoma(bottom), obtained from formalin-fixed paraffin embedded (FFPE) tissue, showing the presence of a frameshift variant (c.1000delC) and consistent with retention of both alleles or the presence of nontumoral admixed cells in the DNA. Reference wild-type sequence is shown for comparison.

**Figure 2:**
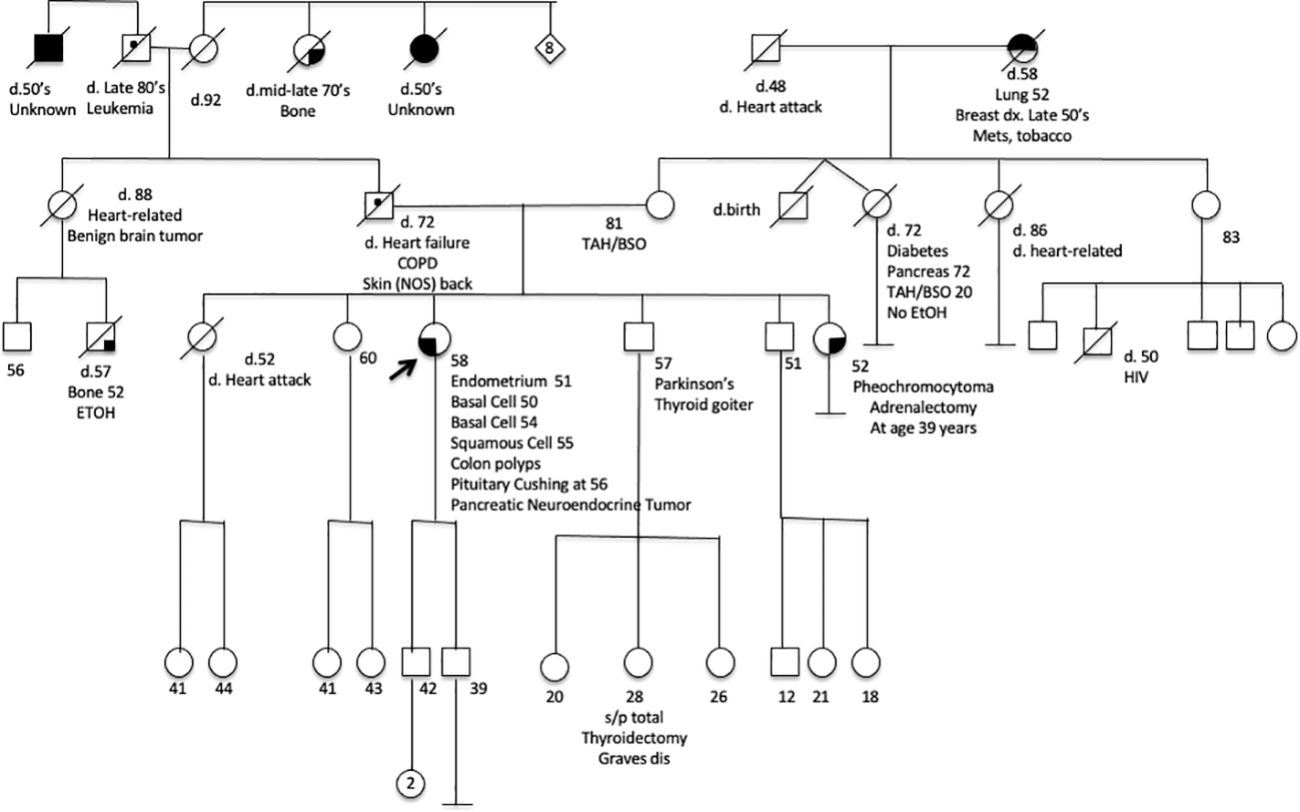
A pedigree chart of the affected family.

58-year old woman with a history of type 2 diabetes, polyneuropathy, colon polyps, hypertension, and obesity was found to have hypercortisolism. Hypercortisolism was suspected when she continued to have rapid weight gain despite following a strict diet and regular exercise. A 1mg dexamethasone suppression test showed an 8AM cortisol of 28 μg/dL (nl<1.8 μg/dL). Her 24 hr urine free cortisol was 89.8 μg/24 hrs (nl<50 μg/24 hrs). The ACTH value was unsuppressed at 69 pg/mL. Pituitary MRI showed a 4mm pituitary adenoma. Inferior petrosal sinus sampling localized the source of ACTH to the pituitary. Patient underwent transphenoidal resection of the pituitary tumor and the histomorphology was consistent with a pituitary adenoma (**Figure 1A**), and immunohistochemical stains confirmed ACTH reactivity. Due to back pain, she underwent a CT scan, which showed a cystic pancreatic mass along the pancreatic body. Fine needle aspiration of this mass was consistent with a PNET. This mass is currently being monitored. Due to patient’s history of pituitary adenoma and PNET, she underwent genetic testing, which revealed *CHEK2* c.1100delC, and no other susceptibility mutation was identified. **Figure 2** depicts her family pedigree, which shows both the patient and her younger sister have the *CHEK2* mutation. We obtained DNA from archival sections of the pituitary adenoma and confirmed the presence of the *CHEK2* mutation, but no evidence of loss of heterozygosity. However, the presence of normal stromal cells may have confounded this analysis.

### Case 2

**Figure 3:**
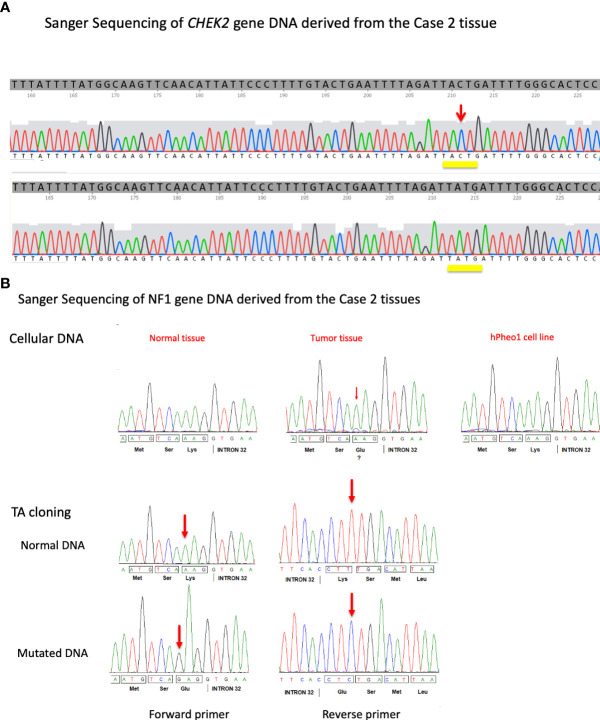
**(A)** DNA sequence traces obtained from the individual clones carrying PCR fragments derived from the hPheo1 cell line derived from the proband’s sister’s pheochromocytoma tumor. Isolates carrying either a wild-type version (*top*) or the mutant version c.1100delC (*bottom*) of the *CHEK2* gene were obtained. The position of the 1100C nucleotide is indicated by a red arrow. **(B)** Formalin-fixed paraffin-embedded tissue DNA from different slides was amplified for *NF1* exon 32 and sequenced along with hPheo1 DNA (*top*). Additionally, PCR products were TA cloned and isolates sequenced (*bottom*). The position of the mutation is indicated (red arrow).

Initially at the age of 39-years old, Case 1’s younger sister was evaluated for nephrolithiasis. At that time, a CT scan detected an incidental 4cm left adrenal mass, which was biochemically confirmed to be a PCC (20) with elevated plasma metanephrines 0.86 nmol/L (<0.50) and normetanephrines 3.09 nmol/L (<0.90). The 24 hr urine metanephrine were 973 mg/24 hrs (<400), normetanephrines 1120 mg/24 hrs (<900), dopamine 200 mg/24 hrs (65–400), norepinephrine 37 mg/24 hrs (15–80), epinephrine 12 mg/24 hrs (0–20). The patient did not have hypercalcemia and had a normal PTH level, without any evidence of other tumors (20). She underwent a left adrenalectomy, and the pathology was consistent with a PCC (**Figure 1B**). The patient was tested for mutations in PCC-associated genes ( (21): *RET*, *TMEM127*, *MAX*, *VHL*, *SDHA, SDHB, SDHC, SDHD*, and *NF1*, and no documented variants were identified. The presence of the *CHEK2* c.1100delC mutation in DNA derived from her PCC and archival PCC tumor tissue was subsequently determined. In addition, using the hPheo1 cell line developed from the patient’s PCC (20), the *CHEK2* locus was analyzed for the presence of c.1100delC using an allele-specific PCR assay using primers Chk2ex10f (5’-TTAATTTAAGCAAAATTAAATGTC), Chk2ex10r (5-GGCATGGTGGTGTGCATC), and Chk2delC (5’-TGGAGTGCCCAAAATCATA). PCR products were separated in 2–3% agarose gels, cloned, and sequencing was used to confirm this mutation (**Figure 3A**). No evidence of loss of heterozygosity was observed. DNA isolated from both the patient’s PCC tumor and hPheo1 cell line derived from it was further analyzed by targeted next generation sequencing and Sanger sequenced and determined to harbor a *KIF1B* variant T827I, considered by PolyPhen-2 to be likely benign. Importantly, this analysis uncovered a pathogenic mutation in the *NF1* gene, NM_001042492.3(NF1): c.4330A>G (p.Lys1444Glu) (**Figure 3B**), associated with predominance of the variant allele (MAF > 70%), and suggestive of loss of heterozygosity of the wild-type allele. These findings support the notion that this *NF1* somatic variant is the driver event underlying this tumor. The patient has not had recurrence fourteen years after adrenalectomy.

## Discussion

In this report, we describe two sisters sharing a *CHEK2* c.1100delC mutation and presenting with endocrine tumors in a pattern not typical of classic multiple endocrine neoplasia. Although it was well known that *CHEK2* mutations are associated with breast and colon cancers, within the last few years, it has been recognized to also be associated with PNETs (8). The first sister does have a PNET. Of note, none of the sisters have a history of primary hyperparathyroidism. In this report, Case 1 had Cushing disease and is being followed for her non-functional PNET. The discovery of her *CHEK2* mutation and the absence of other pathogenic mutations in other susceptibility genes raised the question of whether this variant could be related to this patient’s syndromic phenotype. Pathological studies in Case 1 were not able to show loss of heterozygosity (LOH) in the pituitary tumor sample, as the tumor size was very small and normal stromal cells may have confounded the interpretation of the results (**Supplementary Figure1**).

**Figure 4:**
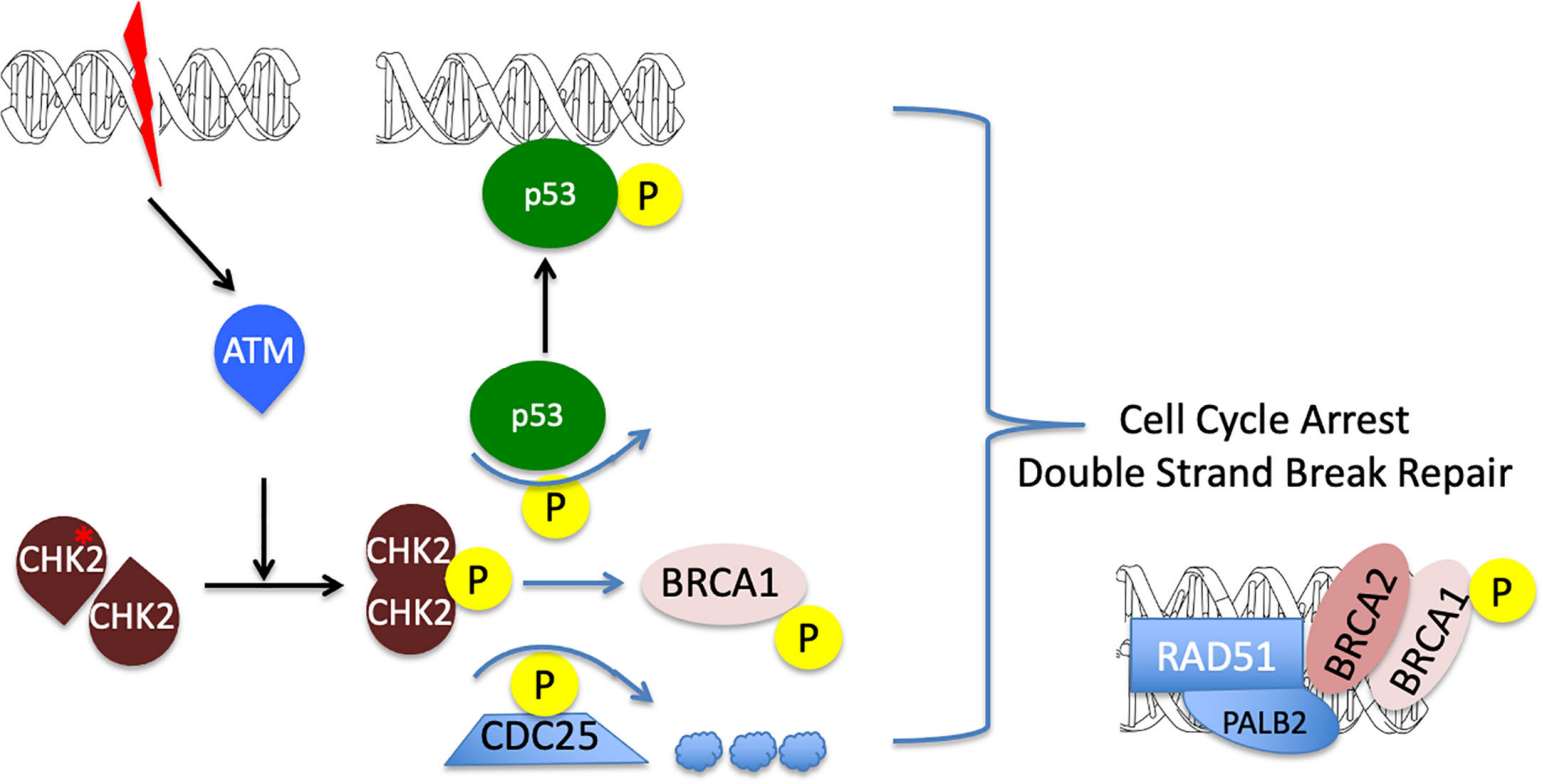
A diagram illustrating CHEK2 function in cell cycle arrest and DNA repair. Double strand break in the DNA leads to ATM activation and subsequent CHEK2 phosphorylation and dimerization. Activated CHECK2 in turn mobilizes p53 and BRCA1 and promotes degradation of the CDC25 phosphatase. The resulting formation of active p53 and RAD51/PALB2/BRCA1/2 DNA complexes, together with a down-regulation of CDC25, leads to the initiation of double strand break repair and cell cycle arrest. Decrease in CHEK2 kinase activity results in the loss of cell cycle control and promotes genomic instability (reviewed in (22)).

The commonality between the pathophysiology of *CHEK2*, and other mutations such as *PALB2* –also associated with PNETs– is that they have an association with DNA repair (**Figure 4**). With DNA damage, CHEK2 is activated, affecting BRCA1/PALB2/RAD51 complex needed for DNA repair (23). Interestingly, Case 1’s younger sister was found to have a PCC and testing of germline from this patient demonstrated the *CHEK2* c.1100delC mutation, but no mutation in PCC susceptibility genes, such as *RET*, *TMEM127*, *MAX*, *VHL*, *SDHA, SDHB, SDHC, SDHD*, and *NF1*. Case 2 did not have any other endocrine tumors such as hyperparathyroidism or medullary thyroid cancer consistent with MEN2 syndrome, or other PCC-related syndromes. As the patient’s tumor produced both metanephrines and normetanephrines, it was predicted that her tumor belongs to the Cluster II classification in PCC genes associated with kinase signaling including *RET, NF1, and TMEM127*, as opposed to Cluster I PCC tumors that are associated with pseudohypoxia signaling pathway (21). Indeed, subsequent evaluation of DNA from the patient’s PCC tumor and hPheo1 cell line detected the pathogenic mutation in the *NF1* gene, NM_001042492.3 (NF1): c.4330A>G (p.Lys1444Glu) (**Figure 3B**). A second variant identified in primary PCC and hPheo1 was *KIF1B* variation T827I (c.2480C > T, rs121908162), which is predicted to be benign by PolyPhen-2 (24). Other mutation, previously reported for hPheo1 line, in addition to *KIF1B*, also includes NRAS Q61K (24) which was not detected in the patient’s tumor DNA, and may represent an adaptation of the cell line in culture (data not shown).

Recently, Dietlein et al. developed an algorithm to identify previously unsuspected cancer driver genes based on the presence of an excess of mutations in unusual nucleotide contexts (25). In their approach, they evaluated whole exome sequencing data from over 11000 tumors of distinct tissue types and identified *CHEK2* as a candidate driver gene in PCCs (25). LOH studies on Case 2’s PCC sample were inconclusive due to the possible presence of normal admixed cells. Therefore, it was difficult to demonstrate that Case 2’s PCC was indeed due to a *CHEK2* driver mutation. The somatic pathogenic *NF1* mutation in Case 2 tumor was associated with a classic second-hit (loss of the wild-type allele), similar to other conventional somatic loss of function *NF1* mutations. Somatic analysis of the pituitary and PNET, which might offer potential insights on somatic events that may have occurred in these tumors, was not feasible. Interestingly, a case in the literature with a different germline *CHEK2* p.R180C with *NF1* mutation has been described. However no endocrine tumors were reported (26).

Future studies with other patients and families will be instructive to assess whether *CHEK2* variants may have any impact as a potential modifier in endocrine neoplasias. Clinically, if a patient has a tumor such as seen in both cases, and genetic testing reveals a germline mutation, the clinician must be aware that the patient’s tumor will need to be sequenced to identify the driver mutation causing neoplastic formation.

## Take-away lessons

In this report, two siblings with a germline *CHEK2* mutation and atypical endocrine tumor associations are described. One sister has a pituitary tumor causing Cushing Disease as well as a PNET. The other sister was found to have a PCC carrying a pathogenic somatic *NF1* mutation. Although all three endocrine tumors (pituitary adenoma, PNET, PCC) can be associated with *NF1*, it was difficult to demonstrate this for the pituitary adenoma and PNET. *CHEK2* is known to be associated with PNET. Whether *CHEK2* mutation could be a contributing factor in a new syndrome of pituitary, PNET, and PCC tumors remains to be determined. Although the clinical relevance of *CHEK2* mutations in these cases cannot be confirmed, we hypothesize that it may play a role in both cases. These two cases underscore the need for resected tumor to be further analyzed, to give a complete picture of whether the germline mutation is solely responsible for tumor formation or there is an additional somatic mutation that serves as a driver. This information can be very important clinically: if the driver mutation is consistent with aggressive disease that would affect the patient’s surveillance and the physician’s ability for early detection of a recurrence with metastatic potential. Solely relying on germline mutation may affect clinical decision-making.

## Patient perspective

### Sister 1

“Now that I am aware of the *CHEK2* variant, I am more diligent with all of my healthcare. I make the recommended cancer screenings a priority. Additionally, since having a pituitary tumor removed, my overall health has significantly improved. I walk four miles a day and have lost 50 pounds. I was able to return to work in the Fall of 2019 and have made great strides in recovering my mental health, affected by Cushings. I have a pancreatic neuroendocrine tumor that is being followed. I have annual CT/MRIs and follow up appointments with the pancreatic oncologist. I believe my experiences with critical and rare health situations has helped me to be more aware of my specific issues and my overall health.”

### Sister 2

“Due to genetic testing I recently found out that I have the *CHEK2* gene. I’m so grateful to have this information because it will most likely save my life. I am now scheduled for several check-ups that I would not have scheduled prior to my genetic testing. My pheochromocytoma was found by accident 14 years ago. Had we known then what we know now my life could be quite different. Genetic testing is a great tool in the business of saving lives.”

## Data availability statement

The variant information has been deposited into ClinVar (www.ncbi.nlm.nih.gov/clinvar) under the accession number SCV002571738.

## Ethics statement

Ethical review and approval was not required for the study on human participants in accordance with the local legislation and institutional requirements. The patients/participants provided their written informed consent to participate in this study.

## Author contributions

RV, YD, KH, JB, SM, AA, ST, MT, OG, PS, PD, HG contributed to the clinical and scientific experiments of the manuscript. RV, YD, KH, JB, SM, AA, ST, MT, OG, PS, PD, HG reviewed the manuscript. HG, ST, OG, PS, PD wrote the initial draft of the manuscript. HG and PD conceptualized the manuscript. All authors contributed to the article and approved the submitted version.

## Acknowledgments

The authors thank the patients and their families. Also, the authors acknowledge Emmanuel Esquivel for his technical assistance with genotyping from the University of Texas Health Science Center at San Antonio. PLMD is supported by NIGMS GM114102, CTSA-IIMS (NIH/NCATS Grant UL1 TR001120 and UL1 TR002645), the Mays Cancer Center NIH-NCI P30 CA54174 and Alex’s Lemonade Stand Foundation (co-funded by Northwest Mutual/Flashes of Hope). HKG is supported by the Gatorade Trust through funds distributed by the University of Florida, Department of Medicine, and Pilot Funding Initiative of the North Florida/South Georgia (NF/SG) VA HS Research Service.

## Conflict of interest

HG has received royalties from the University of Texas Southwestern Medical Center.

The remaining authors declare that the research was conducted in the absence of any commercial or financial relationships that could be construed as a potential conflict of interest.

## Publisher’s note

All claims expressed in this article are solely those of the authors and do not necessarily represent those of their affiliated organizations, or those of the publisher, the editors and the reviewers. Any product that may be evaluated in this article, or claim that may be made by its manufacturer, is not guaranteed or endorsed by the publisher.

## Supplementary material

Supplementary Figure 1 Sanger sequencing traces on *CHEK2* of the proband’s pituitary adenoma, obtained from formalin-fixed paraffin embedded (FFPE) tissue, showing the presence of a frameshift variant (c.1000delC, arrow). Representation of the normal, wild-type (WT) allele and the variant allele are shown, indicating an almost 50-50 distribution, consistent with retention of both alleles or the presence of nontumoral admixed cells in the DNA.
