# Supplementary figures and images for: Case report: Two sisters with a germline CHEK2 variant and distinct endocrine neoplasias
Source: Front Endocrinol (Lausanne). 2022 Nov 7;13:1024108. doi: 10.3389/fendo.2022.1024108 (PMC9682564; doi:10.3389/fendo.2022.1024108)

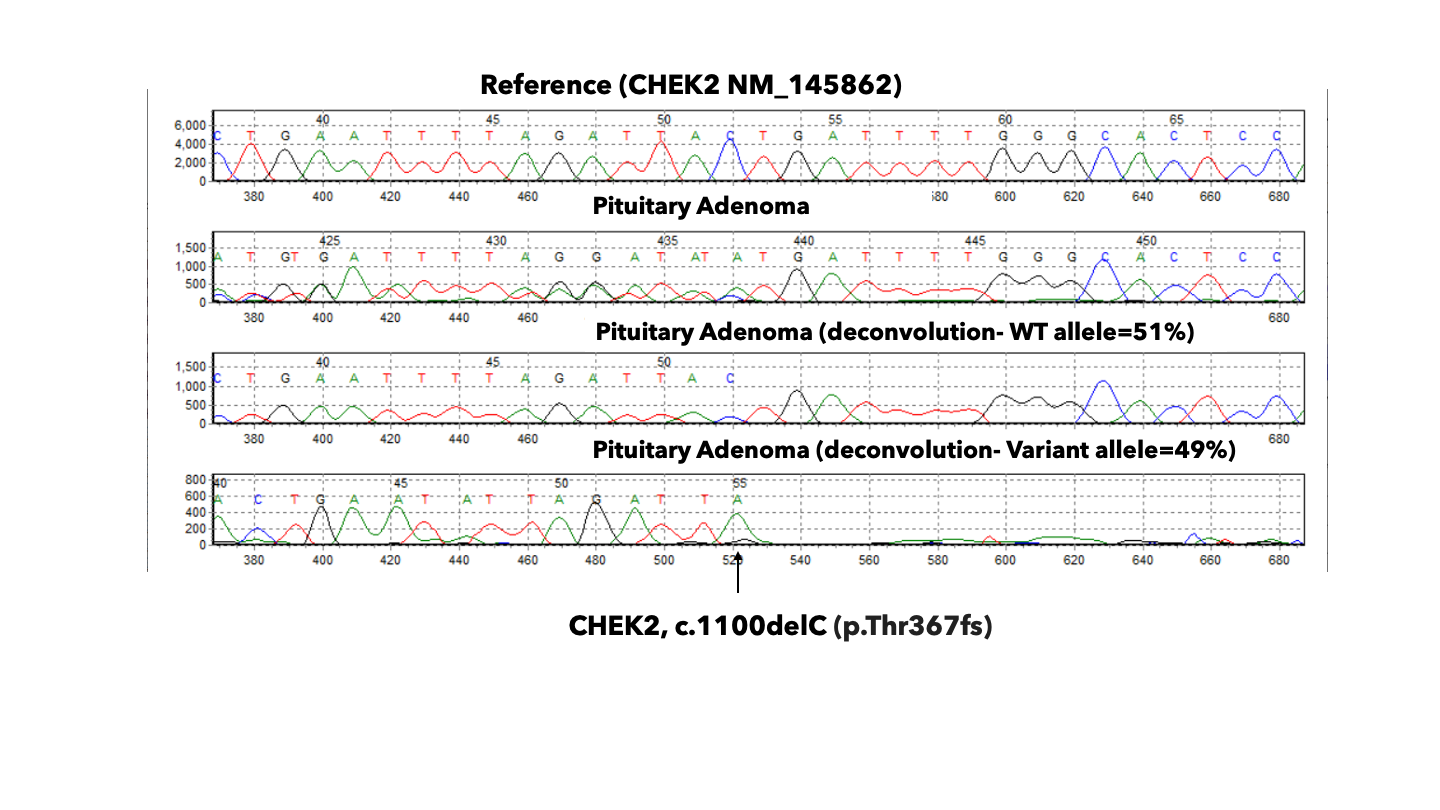

Supplement: Supplementary Figure 1 — Sanger sequencing traces on CHEK2 of the proband’s pituitary adenoma, obtained from formalin-fixed paraffin embedded (FFPE) tissue, showing the presence of a frameshift variant (c.1000delC, arrow). Representation of the normal, wild-type (WT) allele and the variant allele are shown, indicating an almost 50-50 distribution, consistent with retention of both alleles or the presence of nontumoral admixed cells in the DNA. [file Image_1.tiff]
